# Dementia is (not) a natural part of ageing: a cross-sectional study on dementia knowledge and misconceptions in Swiss and Italian young adults, adults, and older adults

**DOI:** 10.1186/s12889-022-14578-8

**Published:** 2022-11-25

**Authors:** Deborah Pacifico, Maddalena Fiordelli, Marta Fadda, Sabatini Serena, Giovanni Piumatti, Fabio Carlevaro, Francesca Magno, Giovanni Franscella, Emiliano Albanese

**Affiliations:** 1 Institute of Public Health, Faculty of Biomedical Sciences, Università Della Svizzera Italiana, Lugano, Switzerland; 2 Fondazione Agnelli, Turin, Italy; 3 Polo Universitario Asti Studi Superiori (Uni-Astiss), University of Turin, Turin, Italy; 4 University of Turin, Turin, Italy; 5 Department of Psychiatry, University of Geneva, Geneva, Switzerland

**Keywords:** Alzheimer’s disease, Knowledge, Attitude, Beliefs, Understanding

## Abstract

**Background:**

Increasing public awareness and understanding of dementia is the second key action area of the 2017 WHO Global action plan on a public health response to dementia. To achieve this aim, the first indispensable step is to understand the average level of dementia knowledge and to identify areas of low dementia knowledge. We aimed to quantify dementia knowledge in the general population, and to explore the extent to which it differs by age, sex, education, and indirect experience with dementia.

**Methods:**

We conducted an online cross-sectional survey in two Italian-speaking sites, south Switzerland (Ticino) and northern Italy (Piedmont). The survey was distributed between September and December 2019. We registered socio-demographic characteristics including whether the participant had contact with a person living with dementia, and measured dementia knowledge with the Dementia Knowledge Assessment Survey (DKAS).

**Results:**

Misconceptions about dementia were common among respondents, and lack of knowledge has been identified in dementia causes, characteristics, risk factors, and health promotion. Our results point out the lack of knowledge about how to communicate and relate with, and take care of a person living with dementia. The overall DKAS score was significantly and positively associated with female sex (*β* = 0.21; *p* < 0.001), educational level (*β* = 0.15; *p* < 0.001) and contact with a person living with dementia (*β* = 0.17; *p* < 0.001), but not with age (*β* = -0.01; *p* = 0.57).

**Conclusion:**

Our results confirmed that general population’s knowledge of dementia is thin. Interventional studies that rely on participatory action research methods are warranted to co-design interventions aimed at improving dementia knowledge and understanding in the public.

## Introduction

Dementia is a public health priority as it affects 55 million people worldwide [1]. Dementia has implications on the quality of life and financial resources of those affected by the disease, their caregivers, and the entire society. As there is no effective treatment for dementia, emphasis must be placed on prevention, timely diagnosis, and care [2]. Low dementia knowledge, including the common misconception that dementia is a normal part of aging, is a major barrier to engagement in preventative behaviors. Indeed, secondary prevention, i.e. early diagnosis and dementia detection, is crucial; a survey conducted in 2005 shows that 81% of respondents do not recognize the difference between dementia early signs and changes due to the normal aging process [3]; this misconception might lead to postponing visits with healthcare professionals. In 2012, the World Health Organization (WHO) called for a public health approach to dementia to reduce its global burden [1]. In 2017, the WHO further elaborated a global action plan on a public health response on dementia which included, as second action point, increasing public awareness and understanding of dementia [4]. Evidence on the average knowledge, awareness, and understanding of dementia is indispensable to inform a public health response to dementia that is tailored and proportionate to the population’s needs in terms of both dementia prevention and support for those who are living with the disease and their caregivers.

The Alzheimer Disease International (ADI) Word Alzheimer Report 2019 [5] poses at its core the results of a global survey focused on behavioral responses to the disease, attitudes towards personal risk, people living with the disease and dementia treatment, and knowledge. Nearly 61 000 people responded to the online survey, that found marked between and within countries heterogeneity but low overall dementia knowledge among responders. This finding is concerning as low dementia knowledge in the three areas covered by the ADI survey may contribute to stigma and prejudice against dementia. The survey identifies low dementia knowledge in Italy and Switzerland, for example, 73.7% and 68.5%, respectively, stated that people living with dementia are impulsive and unpredictable. However, the survey focused primarily on stigma related knowledge, attitudes and beliefs in relation to dementia, and did not rely on previously validated measures of dementia knowledge as a composite construct [5]. Thus, it does not offer a general dementia knowledge score which may be comparable to other studies, and does not formally explore knowledge on pathology, causes, and symptoms.

Other existing studies on the topic also relied on scales with substantial limitations including outdated items, focus on a specific disease (i.e. AD) and/or disease stage, or on a specific healthcare system [6]. The Dementia Knowledge Assessment Scale (DKAS) is instead a 25 items questionnaire designed to measure knowledge of dementia from a biopsychosocial perspective [7–9], thus investigating the neurodegenerative condition and its management considering its effects on body, psychological implications, and social interactions [8]. Compared to other scales (e.g., Alzheimer's Disease Knowledge Scale, ADKS [10]), the DKAS has better psychometric properties [11]. Another strength of the DKAS is that it produces, in addition to an overall score for dementia knowledge, four sub-scores on the syndrome causes and characteristics, related risk and protective factors, care consideration and communication with people with the condition [7, 8, 11]. These make it possible to identify specific areas of low dementia knowledge which should be prioritized in future educational efforts/campaigns. The DKAS has been previously used to assess dementia knowledge among health professionals [12, 13] and caregivers [14], and in the general population [15–17].

Because dementia has a relevant impact at the societal level, knowledge and awareness of dementia is important among all age groups, and not only among older adults who are at greater risk of developing the disease. Evidence suggests that educating children and adolescents towards dementia helps reducing stigma, and thus might improve dementia detection, promote access to, and use of services for diagnosis and care [18–20]. However, comparisons of dementia knowledge among different age groups in the general population are limited, and evidence from Western countries, particularly from Europe, is scarce [21]. Another information that might play a role in dementia knowledge is indirect dementia experiences, as evidence shows its link with increased awareness [22–24]; nevertheless individuals with a parental family history of dementia have limited knowledge on dementia risk reduction [15].

We conducted a cross-sectional survey between two Italian-speaking samples of young adults, adults, and older adults of the general population in Northern Italy and Southern Switzerland. We aimed to estimate and quantify dementia knowledge in the general population, and to formally explore differences by age, sex, education, and comparing participants who had, with those who did not have a previous direct contact with a family member or acquaintance living with dementia.

## Methods

### Study design

We designed, piloted, and tested an online survey on dementia knowledge using a secured electronic data capture system (REDcap software) [25]. We used the survey in a cross-sectional study in two Italian-speaking sites, in Switzerland (Ticino) and Northern Italy (Piedmont). The questionnaire was self-administered in Italian language and data were collected anonymously. All methods were performed in accordance with the relevant guidelines and regulations.

### Sampling and recruitment

The study population included individuals aged 18 years or above, living in one of the two regions of interest, i.e., Ticino and Piedmont. The survey was distributed between September and December 2019. We used convenience sampling following two different recruitment strategies. First, leveraging the collaboration with a local association of older adults in Ticino we distributed the survey through their mailing list and disseminated it through their social media channels (i.e., their Facebook page, which counts 1468 followers). Second, following a snowball sampling procedure, Master students from the “Physical education” and “Communication” Faculties from the two regions completed and circulated the questionnaire across their acquaintances.

### Measurements

The survey started with a brief demographic form (comprising sex, age, education level, nationality, and whether the participant had contact with a person living with dementia) and continued with the Dementia Knowledge Assessment Survey (DKAS) [8]. Overall, the survey takes approximately 15 min to complete. The DKAS comprises a mix of true and false statements about dementia. Participants express their (dis)agreement with each of the 25 statements using a Likert scale that ranges from “false” (1) to “true” (4), through “probably false” (2), “probably true” (3), with the option to select “I don’t know” (5). We followed the original scoring instructions, recoded responses, and computed the DKAS overall score (which ranges between 0 and 50), and the four sub-scores of: “Causes and characteristics”, “Communication and behavior”, “Care considerations”, and “Risk and Health Promotion” [7]. The “Causes and characteristics” subscale focuses on dementia pathology and the course of the disease; the subscale “Communication and behavior” accents how and if a person with dementia relates with others; the subscale “Care considerations” highlights the areas of impairment and thus the symptoms relevant to the provision of care; finally, the subscale “Risk factors and health promotion” explores the knowledge about risk factors and health behaviors associated with the disease. Two independent investigators fluent in both English and Italian translated the original English version of the DKAS into Italian, which were back-translated by a third researcher, and scrutinized for discrepancies. Disagreement was resolved through discussion among team members. Cronbach’s alpha (α) for the overall DKAS in this sample is 0.84 indicating good scale reliability. The “Causes and characteristics” subscale consisted of 7 items (α = 0.59), the Communication and behavior”, “Care considerations”, and “Risk and Health Promotion” subscales consisted of 6 items (α = 0.68, α = 0.78, and α = 0.57, respectively).

### Statistical analysis

We used means and proportions for descriptive statistics of the sociodemographic variables, and Chi squared tests for comparisons across age groups. Young adults comprised participants aged 18–29; adults participants aged 30–59; older adults participants aged 60 years or older. We checked for skewness and kurtosis, which proved to be in the acceptable range. For a sample size > 300, normality of data are determined by the absolute values of skewness (≤ 2) and kurtosis (≤ 4) [26, 27]. QQ plots have also been considered, which show that observed data are approximate to the expected one. We computed the mean DKAS overall and sub-scores, calculated standard errors, and formally tested differences across age groups using univariate ANOVA, and presented the age stratified DKAS scores and sub-scores with box plots. Next, we used independent samples t-test, and multiple linear regressions to explore the association between demographic variables (i.e., age, sex, educational level, and contact with a person living with dementia) and the DKAS total score and sub-scores. We conducted all analyses in SPSS (IBM SPSS Statistics 26.0 [28]), setting level of statistical significance at 0.05, and with two-tailed tests specifications.

### Ethical approval

We submitted our study protocol to the Ethics Committee of the Canton of Ticino, which informed us that our study did not fall within the scope of Art. 2 of the Swiss law on human research. For this reason, the study did not require ethics approval. All participants gave informed consent to participate prior to filling out the online survey. No personal data were collected.

## Results

### Sociodemographic characteristics

Overall, 1500 participants responded to the survey and form the analytic sample. Of them, 62.3% were women, 40.9% were young adults (18 to 30 years old), 11.7% adults (30 to 60 years), 47.5% were older adults (60 years or older), and 9.1%, 54.7%, and 36.2% had a primary, secondary and tertiary educational level, respectively. About one-third (34.9%) of participants had a family member or a friend diagnosed with dementia (Table 1).

**Table 1:** Sociodemographic characteristics of participants by age group, *N* = 1500

	18–29 (*N* = 613)	30–59 (*N* = 175)	≥ 60 (*N* = 712)	Chi-squared test	*p* value^a^
	N (%)	N (%)	N (%)		
**Sex**					
Male	255 (41.6)	52 (29.7)	258 (36.2)	9.37	< 0.01
Female	358 (58.4)	123 (70.3)	454 (63.8)		
**Educational level**					
Primary	13 (2.1)	18 (10.3)	105 (14.7)	151.93	< 0.01
Secondary	417 (68.0)	45 (25.7)	359 (50.4)		
Tertiary (high school or university)	183 (29.9)	112 (64)	248 (34.9)		
**Contact with a person with dementia**					
Yes	416 (67.9)	109 (62.3)	452 (63.5)	3.49	0.18
No	197 (32.1)	66 (37.7)	260 (36.5)		

^a^ *p* values are from Chi Squared tests

### Dementia awareness and knowledge

**Fig. 1:**
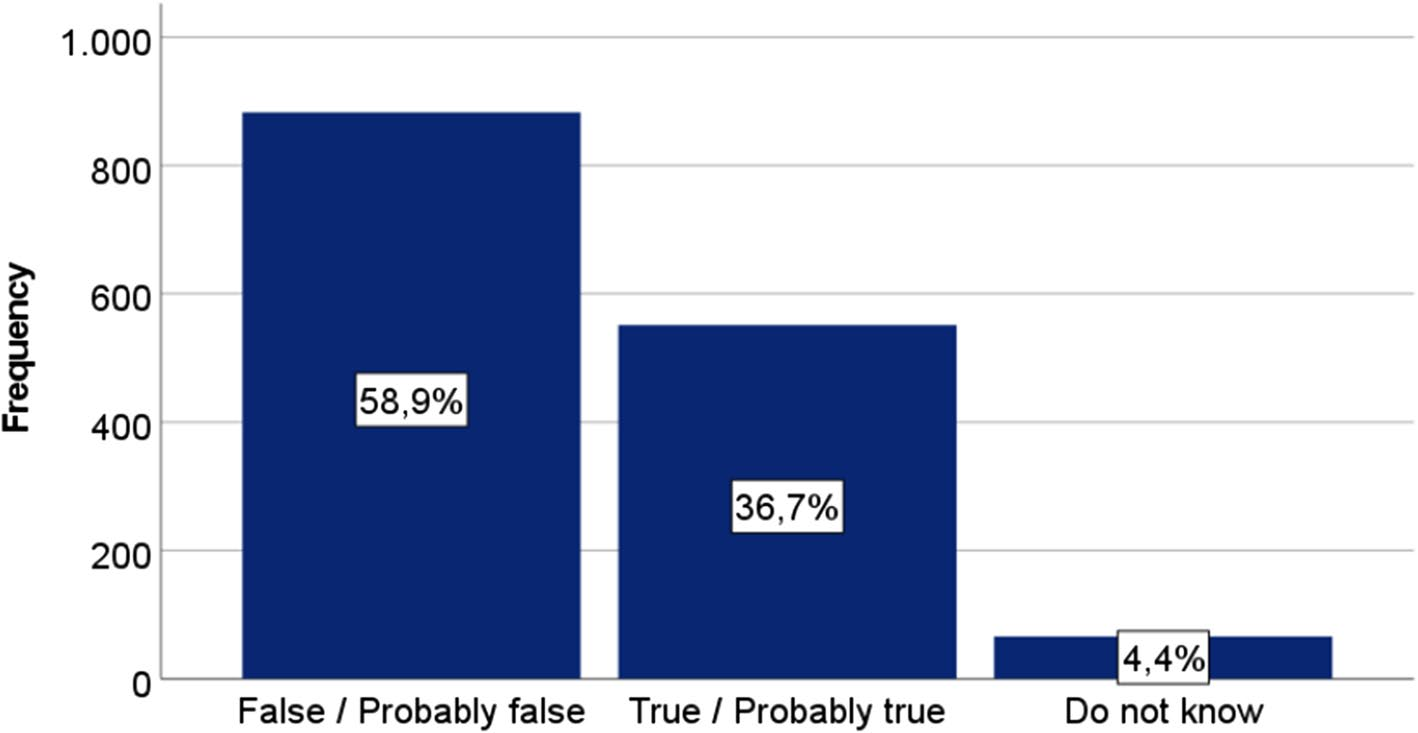
DKAS item one responses: dementia is a normal part of the ageing process

Misconceptions about dementia were common in the study sample, 36.7% of these maintained that dementia is or may be a normal part of the ageing process, 4.4% stated that they do not know, and 58.9% stated that dementia is or may be a pathological condition not invariably linked to old age (Fig. 1).

**Fig. 2:**
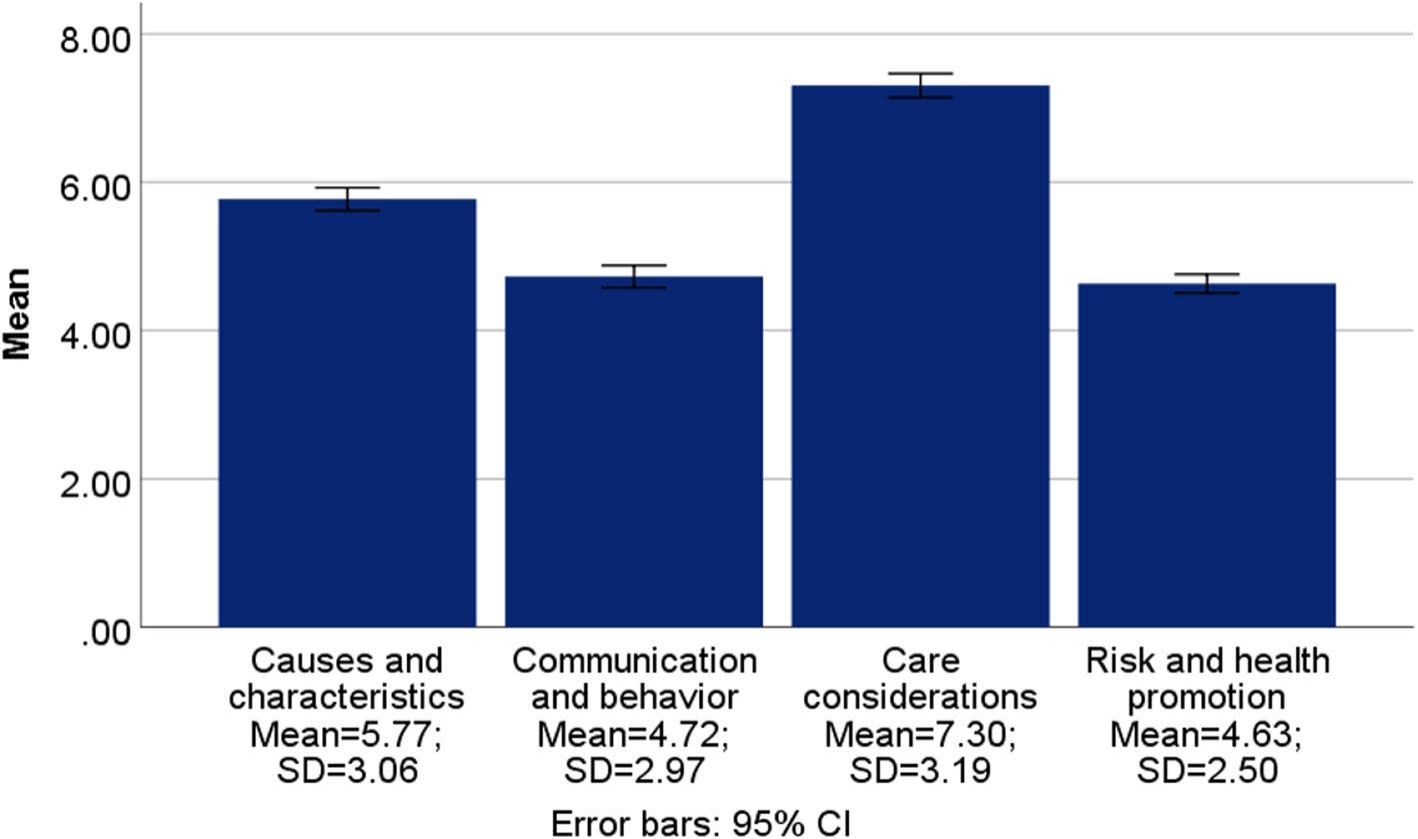
Dementia Knowledge Assessment Scale (DKAS) subscales scores

The DKAS score ranged between 0 (minimum value) and 50 (maximum value) (mean = 22.43; SD = 8.88). The 60^th^ percentile of DKAS scores was chosen to represent a sufficient level of dementia knowledge in the community. This score was calculated to be 30. Comparable approaches for arbitrary cutoffs have been used in the literature [16]. Mean sub-scores were lower for the “Causes and characteristics” (mean = 5.77; SD = 3.06; Range = 0–14), the “Communication and behavior”, and the “Risk and health promotion” (mean = 4.63; SD = 2.5; Range = 0–12) subscales (mean = 4.72; SD = 2.97; Range = 0–12) compared to the “Care and considerations” (mean = 7.3; SD = 3.19; Range = 0–12) subscale Fig. 2.

**Fig. 3:**
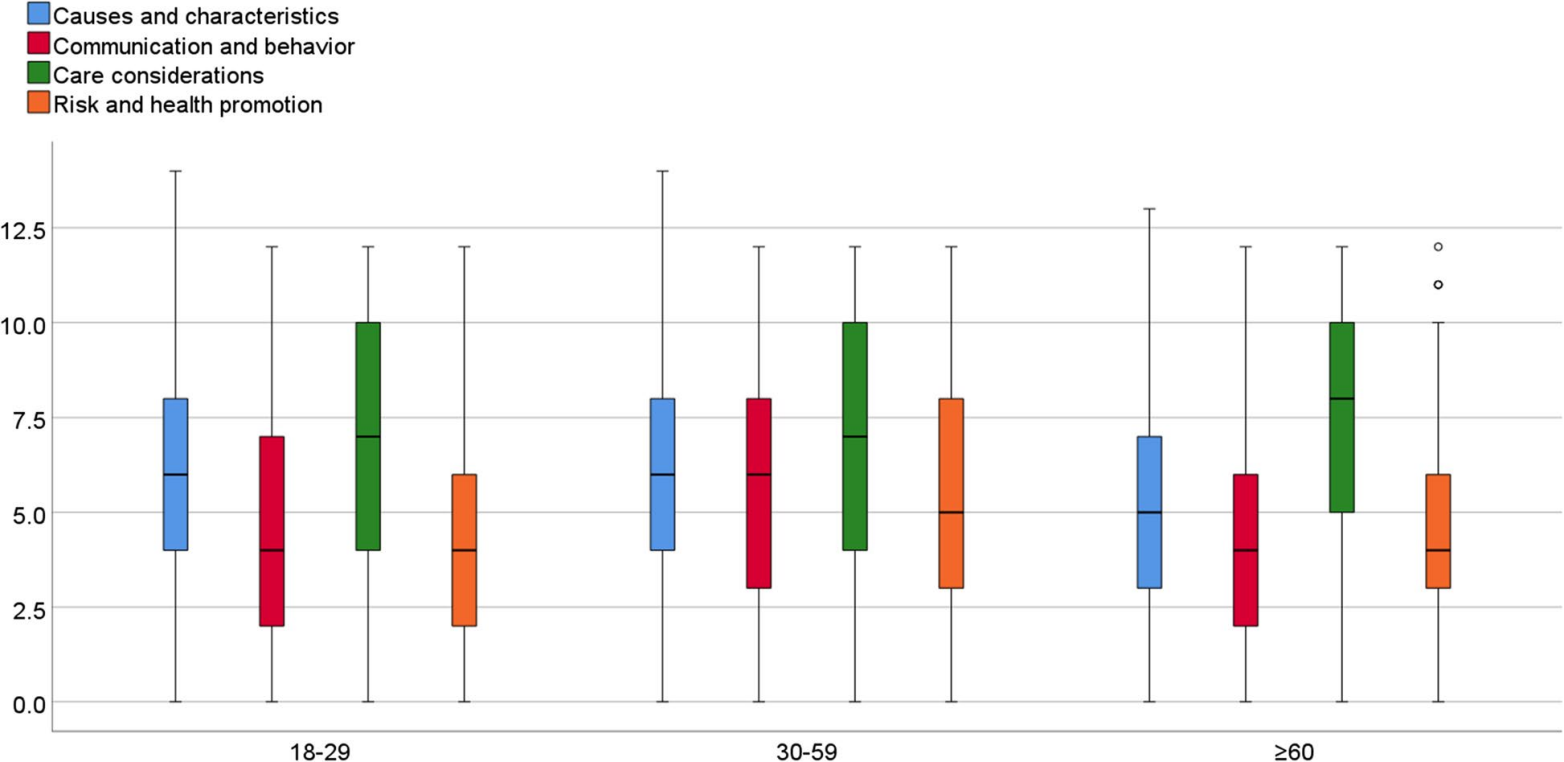
DKAS scores distribution between age groups: 18–29 (*N* = 613), 30–59 (*N* = 175) and ≥ 60 (*N* = 712)

Univariate ANOVA showed that both the overall and subscales dementia knowledge DKAS scores varied across the three age groups (all *p* values < 0.001). Overall, young, and older adults had similar levels of dementia knowledge, and adults aged 30 to 59 years had a higher level of general knowledge about dementia compared to the other groups (*p* value < 0.001). Older adults lacked general and specific (i.e., subscales) dementia knowledge, with the only exception of the care and consideration subscale, on which older adults had highest scores (Table 2, Fig. 3).

**Table 2:** Mean scores, (SD) of dementia knowledge by age group

	Age groups			F statistics (df)	*p* value^a^
	18–29 (*N* = 613)	30–59 (*N* = 175)	≥ 60 (*N* = 712)		
**Overall DKAS score**	22.09 (3.24)	25.21 (10.39)	22.03 (8.03)	9.87 (1499)	< 0.001
**Care and considerations**	6.83 (3.28)	7.23 (3.51)	7.72 (2.98)	13.12(1499)	< 0.001
**Causes and characteristics**	6.28 (3.24)	6.44 (3.40)	5.16 (2.67)	27.75 (1499)	< 0.001
**Communication and behavior**	4.66 (2.85)	5.95 (3.19)	4.48 (2.95)	17.82 (1499)	< 0.001
**Risk and health promotion**	4.31 (2.55)	5.59 (2.86)	4.67 (2.28)	18.61 (1499)	< 0.001

Overall Dementia Knowledge Assessment Scale score possible range: 0 to 50; Care and considerations subscale possible range: 0 to 12; Causes and characteristics subscale possible range: 0 to 14; Communication and behavior subscale possible range: 0 to 12; Risk and health promotion subscale possible range: 0 to 12

^a^ *p* values are based on one-way analysis of variance for continuous variables

We also found that compared to participants who reported not knowing anybody with dementia, those who had a relative or a friend with the condition had a better overall and specific knowledge and understanding of dementia (all *p* values < 0.001). Next, women and participants with a higher educational level had significantly higher scores in all dementia knowledge dimensions compared to men (all *p* values < 0.05) and those with lower educational level (all *p* values < 0.01), respectively.

Consistently, in our multiple adjusted regression model, the overall DKAS score was significantly and positively associated with female sex (*β* = 0.21; *p* < 0.001), higher educational level (*β* = 0.15; *p* < 0.001) and contact with a person living with dementia (*β* = 0.17; *p* < 0.001), but not with age (*β* = -0.01; *p* = 0.57).

Moreover, acquaintance with a person living with dementia, sex, and educational level predicted dementia knowledge across all the DKAS subscales (all *p* values < 0.05), with one exception: the variable sex was not associated with the “Risk and health promotion” subscale. The variable “age group” explained a significant proportion of variance in the “care considerations” (*β* = 0.12; *p* < 0.001), “causes and characteristics” (*β* = -0.18; *p* < 0.001), and “risk and health promotion” (*β* = 0.07; *p* < 0.05) subscales, but not in the “communication and behaviour” one (*β* = -0.04; *p* = 0.13). Table 3 shows multiple regression models for the DKAS overall and subscale scores.

**Table 3:** Linear regression model for DKAS overall score and DKAS subscales

Explanatory variables	R	R^2^ (*p*-value)	Age group, β (SE)	Contact with a person with dementia, β (SE)	Sex, β (SE)	Educational level, β (SE)
**Overall DKAS score**	0.32	0.10 (< 0.001)	-0.01 (0.23)	0.17 (0.46) ***	0.21 (0.45) ***	0.15 (0.35) ***
**Care considerations**	0.31	0.10 (< 0.001)	0.12 (0.08) ***	0.16 (0.16) ***	0.21 (0.16) ***	0.06 (0.13) *
**Causes and characteristics**	0.30	0.09 (< 0.001)	-0.18 (0.08) ***	0.12 (0.16) ***	0.16 (0.16) ***	0.12 (0.12) ***
**Communication and behavior**	0.28	0.08 (< 0.001)	-0.04 (0.08)	0.13 (0.15) ***	0.18 (0.15) ***	0.15 (0.12) ***
**Risk and health promotion**	0.18	0.03 (< 0.001)	0.07 (0.07) *	0.09 (0.13) **	0.05 (0.13)	0.13 (0.10) ***

Standardized Β coefficients from adjusted linear regression models of DKAS overall and subscales scores per age groups (reference: 18-30y), contact with person with dementia (compared to no contact, reference), sex (reference: male), and educational level (reference: primary school)

*R* = Correlation between the observed and predicted DKAS overall and subscales scores

*R*^2^ = Proportion of variance in the DKAS scores explained by the four independent variables included in the linear regression model

^*^, **, *** indicates *p* values < 0.05, < 0.005, < 0.001, respectively

## Discussion

Our study explored dementia knowledge in two Italian-speaking bordering countries. We found that false beliefs on dementia were pervasive, and that dementia knowledge and understanding among young adults, adults, and older adults was low, particularly in the areas of dementia causes and characteristics, risk and protective factors, caregiving and communication. On average participants had low dementia knowledge, as indicated by their means scores on the DKAS, which is lower than the 60^th^ percentile. Variability in levels of dementia knowledge across age groups was noticed. Adults aged 30 to 59 had better dementia knowledge than younger and older adults in most of the dementia knowledge domains investigated. Older people were the most knowledgeable in the area of caregiving and communication. Finally, we found that high educational levels, being a woman, and having or having had direct contact with a family member or acquaintance living with dementia were associated with greater dementia knowledge. The lack of significant association between age and the overall DKAS score might be related to the variability across the investigated dementia knowledge domains.

Our results on higher dementia knowledge levels among women are in line with previous studies [29, 30]. This might be due to the fact that caregivers of people living with dementia are typically women [31, 32], but it might be related to gender differences in health information behavior and health literacy in general: women may be more prone to actively seek information about health-related topics [33]. Similarly, our results on the positive association between educational levels and dementia knowledge are in line with a large body of literature highlighting a positive association between health literacy and education [34].

Our findings are based on the DKAS, which has been used to assess the acquisition of dementia knowledge through educational online courses [35] and interventions [36, 37]. However, so far the DKAS has mainly been used for descriptive purposes in specific populations of informal dementia caregivers [14], medical students [38], and health workforce [13]. Our results extend evidence on low dementia knowledge, as assessed with the DKAS, to the general population in Italy and Switzerland. Our findings are in line with those of other studies that, even though assessed dementia knowledge with different questionnaire, found low dementia knowledge in the general population [23, 29].

Our study explored dementia knowledge in young adults and compared dementia knowledge among young adults, adults, and older adults. Our finding that young adults have lower dementia knowledge than adults aged 30 to 59 has important implications for dementia prevention. Indeed, as estimations suggest that engagement in health-related behaviors could prevent up to 40% of cases of dementia [2] and the benefits of engagement in healthy behaviors are cumulative over time [39], poor knowledge of preventative behaviors amongst young adults could represent a challenge for containment of future cases of cognitive decline and dementia. Notably, midlife is a key moment in the life-course to address vascular dementia risk factors, such as hypertension and obesity [40]; our results on people in midlife knowledge about risk factors is promising, but it is important to foster younger adults’ knowledge on the topic.

Our finding that, compared to adults, dementia risk perception was lower among young adults and older adults suggest that dementia risk perception varies among the life course. Low dementia risk perception in young adults is not surprising because dementia and old age are intimately related. Low levels of dementia risk perception among older adults compared to those aged 30 to 59 may represent an optimistic bias in older adults [41]. Older adults were however more knowledgeable in how to provide care to a person with dementia. This may be explained by a greater direct involvement in informal caregiving in older compared to younger adults [42]. This reasoning is further supported by the higher levels of dementia knowledge we found among those having a family member or acquaintance with dementia.

Age was not associated with knowledge in how to communicate and behave with a person living with dementia, and nearly half of our sample stated that it could be even impossible to communicate with a person who has severe dementia. This common misconception is worrisome and may contribute to increase loneliness and isolation, disease burden [43], and social exclusion [44] among people with dementia. A deeper understanding of dementia, more specifically of how to connect with those living with the condition, is in the roots of a dementia-friendly society, and a prerequisite to enable people with dementia to actively participate in society. Poor knowledge of dementia symptoms and its course can also hamper help seeking, and reduce access to and use of services in older adults.

In sum, consistently with research conducted in other areas of the world, our results highlight a general lack of dementia knowledge and the presence of false beliefs and misconceptions about dementia in Swiss and Italian young adults, adults, and older adults [45]. Hence the general population would benefit from educational interventions on dementia. Our findings that lower educational levels, being a man, and lack of direct contact with a family member or acquaintance living with dementia were associated with less dementia knowledge suggest that, in designing future interventions aiming to increase dementia knowledge, policy makers should take into account the specific target population socio-demographic information, on top of the indirect dementia experience the public may have. As said, our findings are consistent with a large body of evidence that suggests that false beliefs and misconceptions about dementia persist. It is worth noting that ignorance, in its etymological meaning of ‘lack of knowledge’, is in fact not a void or absence of information, but instead a distorted perception of factual knowledge. This implies that scientists and experts have the responsibility to engage with the public in order to disseminate and communicate the results of their research as accessibly and comprehensibly as possible. Communication should be bi- not uni-directional because transfer of knowledge that does not account for existing stances and beliefs cannot substitute ignorance. The participatory action research approach posits that researchers and communities should work in partnership to understand and improve the circumstances they experience [46]. Therefore, interventions aimed at improving dementia knowledge in all age groups may be more likely to contribute to reduce the individual and societal impact of dementia if they entail a theory-based dialogue between experts and the public. Moreover, Dementia may be associated with stigma. Because it cannot be excluded that more and better knowledge of dementia in the general public may contribute to stigma, all actions aimed at increasing dementia awareness must carefully consider and entail parallel efforts to address and possibly reduce stigma as well.

Several limitations of this study should be noted. Although the study sample was large, it was not representative, and our recruitment strategy might have led to selection bias. As we collected data online, older adults with higher education and higher digital literacy may be overrepresented. Second, we did not have access to information about participants’ work in the health sector, which might affect dementia knowledge levels. Moreover, our sample comprised fewer adults aged 30 to 59 years compared to both young and older adults. Besides age, the study sample has a broad sociodemographic spectrum which provides support to internal validity. Nonetheless, our results should be generalized with caution and to similar populations only. The DKAS has not been validated in Italian language, but we strictly followed the WHO recommendations for its translation and lexical adaptation favoring conceptual and cultural rather than literal equivalence [47]. Moreover, differently from previous studies on dementia knowledge that lacked of validated questionnaires [5], we used a validated measure of dementia knowledge. A validation study was deemed unnecessary also because the DKAS focuses on knowledge about dementia under a biopsychosocial perspective that is presumably culturally invariant, that varies more markedly within rather than between countries and contexts. Measuring dementia awareness through the DKAS might be sub-optimal, and it is of paramount importance to explore the nature of dementia awareness and to improve our understanding of its measurability at the population level through qualitative research.

Our results on widespread false beliefs about dementia are highly consistent with those found in other studies including the 2019 ADI survey on dementia attitudes and stigma [5]. This may provide some empirical support of convergent validity. There is, obviously, no gold standard measure to compare the DKAS to. While criterion validity of the DKAS cannot be quantified, construct validity may not be demonstrated either, and assumptions about an hypothetical underlying construct of dementia knowledge may be relapsed, irrespective of measurability.

## Conclusions

Our results confirmed that general population’s knowledge of dementia is thin. Dementia knowledge is indispensable to increase and improve dementia awareness and friendliness, which in turn are very important to limit the barriers to diagnosis and care, and to set the foundation of dementia risk reduction and prevention in the general population. A horizontal participatory process is warranted to co-design interventions aimed at improving dementia knowledge and understanding in the public. This should inform and precede the implementation of these interventions at scale, maximizing their potential to contribute to attain the ambitious goal of reducing the global impact of dementia.

## Data Availability

The dataset generated and analyzed during the current study is available in the Zenodo open access repository, 10.5281/zenodo.6497273.
